# Giant bilateral symptomatic adrenal myelolipomas manifested in an adult with congetnial adrenal hyperplasia

**DOI:** 10.1186/1687-9856-2013-S1-P116

**Published:** 2013-10-03

**Authors:** Yoo-Mi Kim, Jin-Ho Choi, Beom-Hee Lee, Gu-Hwan Kim, Beom-Sik Hong, Yong-Joon Ryu, Han-Wook Yoo

**Affiliations:** 1 Department of Pediatrics, Asan Medical Center Children’s Hospital, University of Ulsan College of Medicine, Seoul, Korea; 2 Medical Genetics Center, Asan Medical Center Children’s Hospital, University of Ulsan College of Medicine, Seoul, Korea; 3 Department of Urology, Asan Medical Center Children’s Hospital, University of Ulsan College of Medicine, Seoul, Korea; 4 Department of Pathology, Asan Medical Center Children’s Hospital, University of Ulsan College of Medicine, Seoul, Korea

## 

Adrenal myelolipoma is an uncommon, non-functioning tumor composed of variable amounts of mature adipose tissue and scattered islands of hematopoietic elements, including erythroid, myeloid, lymphoid series, and megakaryocytes. Diagnosis of adrenal myelolipomas based on radiologic imaging, such as ultrasonography, CT or MRI is effective in more than 90% of cases. It should be differentiated from other fat-containing adrenal masses such as teratoma, lipoma and liposarcoma. The optimal treatment depends on the size and symptoms of the tumor. Surgical treatment is usually not necessary for asymptomatic adrenal myelolipomas smaller than 4 cm. In contrast, symptomatic, complicated, and hormonally active myelolipomas larger than 7 cm should be surgically removed. This report present a 50-year old adult raised as a male with giant adrenal myelolipomas manifested as adrenal masses, which was misdiagnosed with liposarcoma by radiologic examination. The patient has been raised as male despite of ambiguous genitalia and thorough investigation was not performed because of poor socioeconomic status. Physical examination showed short stature (<3rd percentile), hyperpigmentation, and micropenis without palpable gonads. There were uterus and ovary in pelvic cavity on abdominopelvic CT. ACTH stimulation test showed adrenal insufficiency. Steroid replacement was initiated before bilateral adrenalectomy. The histologic findings revealed myelolipomas, and endocrine and molecular investigation lead to the diagnosis of 21-hydroxylase deficiency. Karyotype was 46,XX and mutation analysis of *CYP21A*2 identified a compound heterozygosity consisting of p.I173N and p.Q319*. The patient has been treated with dexamethasone 0.5 mg once daily and fludrocortisone 0.1 mg once daily. As subject has been raised as a male, additional operation such as oophoro-hysterectomy is under consideration. Patients with congenital adrenal hyperplasia should be screened for incidental adrenal masses to avoid unnecessary surgical procedures.

